# Effects of Spaceflight on Cells of Bone Marrow Origin

**DOI:** 10.4274/tjh.2012.0127

**Published:** 2013-03-05

**Authors:** Engin Özçivici

**Affiliations:** 1 İzmir Institute of Technology, Department of Mechanical Engineering, İzmir, Turkey

**Keywords:** Hematopoetic stem cells, Blood cells, Immunity, Stem cell physiology, Lymphocyte, Monocyte

## Abstract

Once only a subject for science fiction novels, plans for establishing habitation on space stations, the Moon, and distant planets now appear among the short-term goals of space agencies. This article reviews studies that present biomedical issues that appear to challenge humankind for long-term spaceflights. With particularly focus on cells of bone marrow origin, studies involving changes in bone, immune, and red blood cell populations and their functions due to extended weightlessness were reviewed. Furthermore, effects of mechanical disuse on primitive stem cells that reside in the bone marrow were also included in this review. Novel biomedical solutions using space biotechnology will be required in order to achieve the goal of space exploration without compromising the functions of bone marrow, as spaceflight appears to disrupt homeostasis for all given cell types.

**Conflict of interest:**None declared.

## Abstract (tr)

Uzay istasyonları, Ay ve uzak gezegenlerde insan yaşantısının sürdürülmesi her ne kadar bilim kurgu romanlarının öğesi olsa da, bugün uzay ajanslarının kısa vadeli hedefleri arasında yer göstermeye başlamıştır. Bu bilimsel derleme, insanoğlunun uzun süreli uzay uçuşları sırasında karşılaştığı biyomedikal problemleri sunma hedefiyle yazılmıştır. Özellikle kemik iliği kökenli kemik, kan ve bağışıklık hücrelerine yoğunlaşılan derlemede bu hücrelerin ağırlıksız ortamda yaşadığı sayısal ve fonksiyonel değişiklikler sunulmuştur. Mekanik kuvvet yoksunluğunun sadece özelleşmiş hücrelerde değil aynı zamanda kemik iliği içinde varolan öncül kök hücrelere olan etkisi de derlemeye eklenmiştir. Özetle, uzay uçuşları kemik iliğinde bulunan bütün hücrelerin düzenini bozduğu için, uzun süreli uçuşlarının sağlıklı gerçekleşme potansiyelinin ağırlıksız ortamın yarattığı ters etkileri ortadan kaldırabilecek yenilikçi biyomedikal çözümler ve uzay teknolojilerine bağımlı olacağı öngörülmüştür.

## INTRODUCTION

Space travel is among the top goals of humankind. Significant progress has been made in spaceflight technologies since the 1960s. These improvements extended the duration of space habitation from minutes to days, months, and even years in some cases [1]. Human activity beyond the low orbit is not expected to cease, as prospective plans for long-duration space missions to the Moon and Mars are in action [2]. From a purely technical standpoint, the duration of a spaceflight is a problem of logistics that requires careful optimization of escape trajectories with required fuel and sustenance. However, the biological response of astronauts (and cosmonauts] to space, an environment in which spaceship crew constantly experience weightlessness, presents equally challenging and unique biomedical problems for the duration of spaceflight missions. These challenges need to be addressed for humans to travel, live, and work in space and on distant planets.

The human adaptive response to weightlessness encompasses numerous conditions that may affect the possibility of long-term flight missions [3]. These conditions include space motion sickness, cardiovascular deconditioning due to reduced blood volume, and prolonged gastrointestinal transit time. Moreover, the immune system is known to be suppressed while bacterial pathogens appear to be unaffected by, if not benefiting from, spaceflight conditions. Severe and progressive catabolism in space also affects the musculoskeletal system. Spaceship crew members progressively lose bone minerals from weight-bearing sites, increasing their susceptibility to kidney stones (nephrolithiasis] during flight as well as traumatic or non-traumatic fracture both during and after the flight mission. Loss in bone mass is also accompanied with significant losses in leg muscle volumes caused by muscle atrophy. 

Mission fidelity and optimum quality of life for spaceship crew is endangered by these medical conditions. Prophylactic and/or treatment strategies to alleviate these adverse conditions are the subject of active research in space biomedicine. Furthermore, even though some of these conditions are transient and recovery is observed after returning to regular weight-bearing activities, some others may remain persistent for the crew. Given that bone turnover activity and cellular constituents of the immune system are actively regulated by progenitor/stem cells that reside in the bone marrow, adverse effects of disuse caused by weightlessness on these cells have to be analyzed carefully in order to develop strategies to improve spaceflight periods and even make it possible for spaceship crew to populate other planets or satellites. The goal of this review is to present up-to-date biomedical research addressing the response of bone marrow cells to disuse caused by loss of weight-bearing. 

**Mechanical Loads in Biology**

Since life has evolved in the presence of 1 g (‘g’ being Earth’s gravitational pull, which is a constant acceleration of 9.81 m/s^2^] for the last couple of billion years without any interruption in the gravitational field, all cells are adapted to survive and thrive in the presence of mechanical signals. Mammalian cells, similar to plant [4], fungal [5], and bacterial cells [6], can detect and adapt to mechanical forces [7]. The source of these physical forces can be both external (atmospheric pressure, sea waves, wind, etc.] and internal (weight occurring from gravity, blood pressure, interstitial fluid shear, etc.] for organisms. Since mechanical forces are omnipresent in the environment, cells can base numerous decisions about proliferation, migration, commitment, matrix synthesis, and maintenance on the mechanical inputs from their environments [8]. Based on these decisions made by corresponding cells, tissues may follow by altering their form and function. For example, individual muscle fiber (sarcomere] contractions and accumulated damage during physical exercise triggers events that eventually increase the muscle tissue mass of an athlete, a process that would make the athlete physically stronger. Conversely, because of the reduction of mechanical loads during spaceflight, spaceship crew members constantly lose bone mineral from weight-bearing sites of the skeleton [9]. This adaptive response of cells to the presence and absence of mechanical loads needs to be fully understood in order to foresee and prevent negative effects of sustained weightlessness experienced on long-term spaceflights.

**Cellular Niche in the Bone Marrow**

Protected within a calcified cortex, bone marrow houses different types of cells from various developmental backgrounds with absolute importance to the survival of the organism. Other than the small fraction of endothelial cell lineage that primarily forms the marrow vasculature, cells in the bone marrow can be compartmentalized into 2 types with respect to their origins, as mesenchymal and hematopoietic cells. 

Mesenchymal cells can mainly be found in bone, muscle, cartilage, and fat tissues, and they come from a common ancestor, the mesenchymal stem cells. In the bone marrow, mesenchymal stem cells can act as osteoprogenitors, with appropriate endocrine, paracrine, and/or autocrine signals [10]. Osteoprogenitors can drive bone formation by transforming into osteoblasts, the cells responsible for the growth, maintenance, and repair of bone tissue. Mesenchymal stem cells were also shown to commit to the lineages of other mesenchymal tissues such as cartilage, fat, marrow stroma, liver, kidney, and muscle cells [11,12,13,14].

Hematopoietic cells, on the other hand, are non-adherent and constitute cells of lymphoid (B cells, T cells, etc.) and myeloid (granulocytes, macrophages, megakaryocytes, etc.) origins [15]. These cells descend from a common progenitor called the hematopoietic stem cell. In the bone marrow, hematopoietic stem cells generally position (and home) themselves to the proximity of cells of mesenchymal origin [16], highlighting the communicating and regulatory behavior between mesenchymal and hematopoietic cells. 

In a healthy individual, processes such as regeneration, nutrient exchange, metabolite storage, and protection from pathogens are optimized via integrated functioning of marrow cells. Conditions that partially or completely remove mechanical loads on the bone and the bone marrow tissue, such as aging, obesity, bed rest, and spaceflight, adversely affect the marrow cell populations and their functions. Furthermore, the gross composition of the marrow irreversibly changes from red (hematopoietic) to yellow (fatty), indicating a potentially greater scale of disruption in homeostasis. It can be argued that, collectively, these alterations in bone marrow tissue may increase the incidence of morbidity and may even affect longevity for astronauts during long-duration space missions.

**Bone Cells and Spaceflight**

Perhaps the most prominent function of mesenchymal cells in the bone marrow is to contribute to bone formation. Once adequate exogenous signals are received (biophysical and/or biochemical), mesenchymal stem cells commit to osteoblastic lineage. Lining on the calcified tissue, osteoblasts are responsible for new bone formation by attracting calcium ions [17,18]. During this mineralization phase, osteoblasts become trapped in the osteoid lacunae they had been building and transform into osteocytes, a cell type that constitutes the biggest fraction of cells within the bone tissue with important regulatory functions in bone remodeling [19,20]. Parallel to new bone tissue formation, existing bone tissue is actively resorbed by osteoclasts, a large and multinucleated cell type. Osteoclasts come from a monocyte-macrophage origin, and once activated, they facilitate the resorption of the calcified tissue using extracted components with low pH, leading to a net effect of bone loss if their work is not complemented with osteoblasts [21]. Osteoblasts rapidly migrate into perfusions made by osteoclasts and initiate bone formation. Overall, this entire coupled cycle of formation and resorption is called bone turnover. By using this dynamic turnover process, bone tissue is able to: 1] repair a damaged matrix to maintain its strength, 2] adapt to physical forces by adding more bone in the areas of high loading, and 3] act as an endocrine organ to regulate circulating Ca2+ molecules. 

Several conditions that induce reduction in or absence of weight-bearing on long bones influence the coupling between bone formation and resorption, causing osteopenia and eventually osteoporosis [22]. Being either temporary or permanent, these conditions include aging, sedentary lifestyles, confined bed rest, partial paralysis, and spaceflight. During spaceflight, bone mineral is constantly being lost from the weight-bearing sites of the skeleton at an average of 2%-3% per month, accompanied by increases in urine excretion of Ca2+ and hydroxyproline expressing the net bone loss at the tissue level [9,23,24]. Some astronauts were even observed to lose up to 20% bone mass during their missions [25,26], and currently no known plateau exists for this disuse-induced bone loss occurring during spaceflight [27]. Experimental evidence shows that bone formation activity is suppressed during spaceflight at the tissue level [26,28,29], preventing the effective recovery of lost bone tissue for spaceship crew. 

On the cellular level, osteoblasts show response to actual (in-flight) or simulated weightlessness conditions. Osteoblast proliferation was found to be curbed during weightlessness in parallel with reduced osteoblast metabolism [30]. The inner morphology of osteoblasts is sensitive to weightlessness, as significant alterations in nucleus shape and size were observed during mechanical unloading [31]. Moreover, osteoblasts may suffer from programmed cell death during disuse, effectively reducing their number [32,33]. Other indicators suggest that osteoblasts increase the secretion of chemical factors that enhance osteoclast recruitment [34], thereby stimulating bone resorption process. While some studies did not find any correlations between weightlessness and osteoclast activity [35,36], others showed increased osteoclast recruitment and pit formation during disuse [37,38]. Regardless, it is expected that osteoclastic activity be in tune with osteoblastic activity, as osteoblasts tightly control osteoclast maturation and activity [39].

**Mesenchymal Stem Cells and Spaceflight**

Mesenchymal stem cells (MSCs), which reside in the bone marrow, are the main source of osteoprogenitors and osteoblasts. However, these primitive cells may lose their commitment to osteogenic lineage and commit to adipogenic lineage as a result of loss in mechanical loads [40,41]. Simulated disuse was also shown to decrease the size and functionality of the marrow mesenchymal progenitor pool, thereby adversely affecting the regeneration of the bone tissue [35]. Once the marrow pool of MSCs is lost due to extended weightlessness, it may not be possible for an individual to retain healthy function once returned to regular weight-bearing conditions [41,42,43]. Because of impaired bone formation, regeneration of tissue that was lost during disuse is often slow and incomplete [44]. Since reintroduction of regular mechanical loads is not capable of fully restoring bone tissue, it is imperative for biomedical research to prevent or treat disuse-induced loss of osteogenic potential in the bone marrow, not only to prevent spaceflight mission related injury, but also to protect the quality of life beyond the mission.

**Blood Cells and Spaceflight**

Resident hematopoietic cells in the bone marrow serve as important contributors of erythropoiesis, myelopoiesis, lymphopoiesis, and bone turnover. Evidently, the functioning of hematopoietic cells is not exempt from the adverse effects of spaceflight. Not only does the bone turnover favor catabolism for spaceship crew, but the red blood cell volume is also reduced with an apparent suppression of the immune system [3].

Coming from a myelopoietic origin, red blood cells mature from erythroblasts and have a high turnover rate as they are exposed to severe mechanical stress during circulation [45]. As a result of spaceflight, spaceship crew members lose around 15% of their red blood cell mass over the course of a few weeks, a condition known as “space anemia” [1,46,47]. The loss of red blood cell mass is due to a physiological process called neocytolysis, in which immature blood cells are selectively hemolyzed because of reductions in the plasma erythropoietin levels [48,49]. Significant reductions in the in vitro maturation of hematopoietic cells to red blood cells were also observed in response to weightlessness [50]. During spaceflight conditions, red blood cells were shown to proliferate less and appeared apoptotic, even in the presence of stimulant factors, further contributing to the “space anemia” phenotype [51]. 

There is strong evidence that spaceship crew members suffer from suppression of the immune system, and the magnitude of this effect may be related to the length of exposure [3]. Abnormalities in the immune system lead to compromised defense against both exogenous and endogenous pathogens, as well as reduced monitoring against aberrant host cells. Unfortunately, experimental observations to date are not conclusive due to small sample size and variable response. However, observed patterns warrant further attention to clarify net effects of long-term spaceflight on the immune system in order to potentiate human habitation of the Moon, space, and distant planets. 

Immune cells that are capable of phagocytosis appear to be affected by spaceflight conditions, as well. The number of polymorphonuclear leukocytes in circulation was repeatedly observed to be increased by 1.5-fold to 2-fold in both short-term and long-term flights [52,53,54,55]. Circulating monocytes were also observed to be increased in numbers after spaceflight [53,55]. However, the phagocytic capacity and oxidative burst potential of neutrophils and monocytes were both found to be significantly reduced in astronauts due to long-term spaceflights, indicating a delay in function for host defense cells against invading pathogens [56,57,58]. 

T lymphocytes originate from a common lymphoid progenitor (Lin^-^, IL-7R^-^, Sca-1^low^, c-kit^low^) in the bone marrow and mature in the thymus gland to facilitate cell-based immunity [59,60]. T lymphocytes have 2 major subpopulations: helper T cells (CD4^+^) that direct other immune cells by secreting cytokines, and cytotoxic T cells (CD8^+^) that directly kill infected or cancerous cells. Previous observations suggested that the number of circulating T cells was reduced during spaceflight for both humans and rodents [52,55,61,62]. Furthermore, interleukin-2, which is a biomarker for T cell activity, was found to be decreased after spaceflight for both helper and cytotoxic T cells [^52^,^62^]. Activation response of T cells to stimulating agents such as phytohemagglutinin was also found to be diminished as a result of spaceflight [62,63].

B lymphocytes also come from the common lymphoid progenitor in the bone marrow and mediate the humoral immune response [59]. Limited data suggest that the matured B cell fraction was significantly suppressed in rodents that were exposed to spaceflight, but no functional data are available for the observed phenotype. The decrease in B cells was found to be accompanied by an increase in the natural killer cell fraction, a large and granular cell type that is responsible for surveillance of cancer cells [62]. 

On a side note, unlike immune cells that are apparently impaired in number and function during spaceflight, bacterial cells may not be affected at all by the weightlessness environment. On the contrary, several strains, including Salmonella enterica and Escherichia coli, were found to benefit from spaceflight [64,65]. Bacterial resistance to antibiotics was also found to be increased [66], with records showing higher rate of mutation accumulation [67]. Unfortunately, artificial environments such as space ships contain many components in which bacteria and mold can thrive, pointing to an increased hazard rate that may be potentiated during long-term spaceflights [68]. Not only the bacterial pathogens but also the latent endogenous viruses, such as Epstein-Barr and Cytomegalovirus, were found to be reactivated during spaceflight, possibly due to the environmental stresses, increasing the risks of infection and cancer for the spaceship crew [69,70,71]. 

**Hematopoietic Stem Cells and Spaceflight**

Accumulated evidence of the alterations in the red and white blood cell population size and functioning associated with spaceflight may have an array of contributors, including loss of mechanical loads, flight stress, diet, and nutrition. It is also entirely possible that the bone marrow stem cells that are responsible for the replenishment of blood cell types are affected by spaceflight conditions and alter the blood cell phenotype. Hematopoietic stem cells are the self-renewing source of all lymphoid and myeloid cells of an organism and they reside in the bone marrow, where the most primitive hematopoietic stem cells seek and reside in the close proximity of the osteoblasts [16]. Bone marrow-derived CD34^+^ cells, which are assumed to be early hematopoietic progenitors, showed reduced proliferation rate due to slower cell cycle progression when exposed to simulated weightlessness without losing their capacity for self-renewal [72]. Furthermore, CD34+ cells appeared less attracted to stromal cell-derived factor, a chemical agent that stimulates the migration of early hematopoietic cells [73,74]. Ultrastructural properties of CD34^+^ cells reflected this reduced chemotaxis, as expressed with reduced cytoskeletal F-actin expression [73]. Functional characteristics of early hematopoietic progenitors were also found to be affected, as observed in the altered maturation to erythrocytes, granulocytes, and macrophages [50,73]. 

Actual spaceflight and simulated weightlessness data from hematopoietic stem cells (HSCs) are limited compared to data from experiments involving other cell types (such as osteoblasts or T cells), as recognition of these primitive cell types in the bone marrow is a recent development [16,75]. However, HSCs are expected to be affected by weightlessness as they were shown to sense and respond to the presence of mechanical loads [76]. Another important aspect of HSC response to weightlessness is the interaction with mature osteoblasts. Osteoblasts support HSCs in vivo [77,78], and the ablation of osteoblasts in the bone marrow induces extramedullary hematopoiesis [79]. Furthermore, osteoblasts were shown to be important mediators of B cell and megakaryocyte differentiation [80,81]. During spaceflight, trabecular bone tissue that has a large surface area is lost from the distal and proximal locations of metaphyses, not only accounting for the loss of bone mass but also affecting HSC populations, as trabecular bone is an active spot for hematopoiesis. Furthermore, disuse-induced loss of trabecular bone is often accompanied by increased adipocyte accumulation in the bone marrow, a condition that can further damage the HSC functioning [82,83]. 

**Conclusions**

In summary, loss of gravitational loads induces adverse effects on different cell types of bone marrow origin, including connective tissue and immune system cells. Furthermore, not only the committed functional cells but also primitive stem cells seem to be affected, jeopardizing the health of spaceship crew on long-term flight missions. This biological response to long-term loss of mechanical loads appears to be a limiting factor for human habitation beyond Earth. Certainly, scientific developments in the field of space and gravitational biology are required to fully understand biological mechanisms that regulate the organism’s response to spaceflight. In parallel, the fields of bioengineering and space biotechnology are in search of the identification and delivery of “physiologically relevant” mechanical loads that would reconstitute homeostasis in the bone and marrow environment [84]. The replacement of mechanical loads may be in the form of repeated bouts to be applied in certain periods to protect osteoblasts, and potentially other cells of the bone marrow, from the adverse effects of spaceflight. In any case, the goal of humankind to utilize space as the final frontier is clear, and technological advances will help to boldly go where no man has gone before.

**Acknowledgments**

This work was kindly supported by the Scientific and Technological Research Council of Turkey (111T577 and 111M604). Critical reviews from Drs. Özden Yalçın Özuysal and Gülistan Meşe from the Molecular Biology and Genetics Department of İzmir Institute of Technology are gratefully acknowledged. 
